# Comparison of gingiva‐derived and bone marrow mesenchymal stem cells for osteogenesis

**DOI:** 10.1111/jcmm.14632

**Published:** 2019-09-10

**Authors:** Quan Sun, Hidemi Nakata, Maiko Yamamoto, Shohei Kasugai, Shinji Kuroda

**Affiliations:** ^1^ Department of Oral Implantology and Regenerative Dental Medicine, Division of Oral Health Sciences, Medical and Dental Sciences Track, Graduate School of Medical and Dental Sciences Tokyo Medical and Dental University Tokyo Japan

**Keywords:** bone marrow, CD90, gingiva, mesenchymal stem cells, osteogenesis

## Abstract

Presently, bone marrow is considered as a prime source of mesenchymal stem cells; however, there are some drawbacks and limitations. Compared with other mesenchymal stem cell (MSC) sources, gingiva‐derived mesenchymal stem cells (GMSCs) are abundant and easy to obtain through minimally invasive cell isolation techniques. In this study, MSCs derived from gingiva and bone marrow were isolated and cultured from mice. GMSCs were characterized by osteogenic, adipogenic and chondrogenic differentiation, and flow cytometry. Compared with bone marrow MSCs (BMSCs), the proliferation capacity was judged by CCK‐8 proliferation assay. Osteogenic differentiation was assessed by ALP staining, ALP assay and Alizarin red staining. RT‐qPCR was performed for ALP, OCN, OSX and Runx2. The results indicated that GMSCs showed higher proliferative capacity than BMSCs. GMSCs turned more positive for ALP and formed a more number of mineralized nodules than BMSCs after osteogenic induction. RT‐qPCR revealed that the expression of ALP, OCN, OSX and Runx2 was significantly increased in the GMSCs compared with that in BMSCs. Moreover, it was found that the number of CD90‐positive cells in GMSCs elevated more than that of BMSCs during osteogenic induction. Taking these results together, it was indicated that GMSCs might be a promising source in the future bone tissue engineering.

## 1 INTRODUCTION

Mesenchymal stem cells (MSCs) refer to the multipotent stromal cells which are present in many adult tissues and play critical roles in tissue healing and regeneration. These cells can differentiate into a variety of cell types, including osteoblasts, adipocytes and chondrocytes in the presence of some stimulations.^1^ Because of their self‐renewal and multi‐lineage differentiation ability, MSCs are promising sources of progenitor cells for tissue engineering and regeneration.^2^

MSCs can be derived from many adult tissues such as bone marrow (BM)^3^ adipose tissue^4^ dental pulp^5^ periodontal ligament^6^ or gingiva.^7^ Although BM was the original source of MSCs the bone marrow mesenchymal stem cells (BMSCs) still have the most frequent utilization in cell therapy applications. Despite BMSCs have been considered the gold standard in cell therapy applications there are still many drawbacks such as extremely low cell yield limited self‐renewal capacity and differentiation potential high invasive harvesting processes and risk of infection at the donor site.^8^

Gingiva is part of the soft tissue lining of the mouth which surrounds and protects the teeth.^9^ In the gingiva, there are some unique structures which are essential for wound healing and repair. In recent years, gingiva‐derived MSCs (GMSCs) have been isolated and characterized from the gingiva which shows promising regenerative and immunomodulatory properties.^7^, ^10^, ^11^ In contrast to other mesenchymal stem cell sources, GMSCs are abundant and easy to obtain through minimally invasive cell isolation techniques.^12^ In addition, some previous studies have revealed that GMSCs showed remarkable tissue reparative/regenerative potential for many different fields, such as skin wound repair,^13^ periodontal regeneration^14^ and tendon regeneration^15^ and also bone defects regeneration.^16^

Indeed, in a previously published work, it was shown that GMSCs were superior to BMSCs for cell therapy in regenerative medicine.^12^ However, a head‐to‐head comparative study addressing their osteogenic differentiation potential in vitro is still missing. Besides, the further research on the mechanism in the phenomenon should be investigated. In this study, we isolated and characterized the mouse gingiva‐derived stem cells for MSCs properties in detail and compared the proliferation rate and the osteogenic differentiation potential of GMSCs and BMSCs. Moreover, the mechanism in the phenomenon that GMSCs showed higher osteogenic potential than that of BMSCs is investigated. The data presented in this study provide evidence towards understanding the biological characteristics of GMSCs that could be used as a good choice when bone tissue reconstruction is needed.

## 2 MATERIALS AND METHODS

### 2.1 Isolation of cells and cell culture

Gingiva‐derived mesenchymal stem cells: Gingivae were collected from the alveolar bone of 6‐week‐old female imprinting control region (ICR) mice, following pentobarbitone sodium euthanasia. Gingivae were placed in sterile phosphate‐buffered saline (PBS, Wako Chemical) at 4°C immediately. The gingivae were then minced into 1‐2 mm^2^ fragments and digested in 0.1% (w/v) collagenase (type I collagenase; Sigma‐Aldrich) in 30 mL PBS at 37°C for 30 minutes. The cell suspension was then centrifuged at 1000 × *g* for 5 minutes to pellet the GMSCs. The cell pellet was resuspended in α‐MEM (Invitrogen) containing 10% foetal bovine serum (FBS; Sigma‐Aldrich) and 100 U/mL of penicillin‐streptomycin (Pen‐Strep; Sigma‐Aldrich) and plated on 60‐mm culture dishes. Cells were incubated at 37°C in a humidified atmosphere consisting of 95% air, and 5% CO_2_ until confluence was reached.

Bone marrow mesenchymal stem cells: Mouse bone marrow cells were flushed out from femurs using a 27G needle and centrifuged at 1000 × *g* for 5 minutes; they were then washed with PBS and centrifuged again. The harvested cells were cultured in a‐MEM containing 10% FBS with 100 U/mL Pen‐Strep until they reached confluence. The medium for both types of cells was refreshed every 3 days.

The third passages of GMSCs and BMSCs were seeded in 24‐well plates (Corning) at an initial density of 5 × 10^4^ cells/well. They were then cultured for 14 days in osteogenic medium supplemented with α‐MEM containing 10% FBS, 100 μmol/L dexamethasone, 50 ng/mL ascorbic acid (Wako Chemical) and 10 m mol/L β‐glycerophosphate (Sigma‐Aldrich). Cells were subjected to osteogenic analyses by measuring alkaline phosphatase (ALP) activity, histological staining (ALP and mineralized nodule staining) and real‐time reverse transcription polymerase chain reaction (RT‐qPCR).

### 2.2 Multipotent differentiation of gingiva‐derived mesenchymal stem cells

#### 2.2.1 Osteogenic differentiation

To induce osteogenic differentiation, the third passages of GMSCs were seeded in 24‐well plates at an initial density of 5 × 10^4^ cells/well. They were then cultured in osteogenic medium supplemented with α‐MEM containing 10% FBS, 100 μmol/L dexamethasone, 10 mmol/L β‐glycerophosphate and 50 ng/mL ascorbic acid for 21 days with medium changes every 72 hours. At day 21, calcium formation was detected by Alizarin Red S staining (Sigma‐Aldrich).

#### 2.2.2 Adipogenic differentiation

To induce adipogenic differentiation, the third passages of GMSCs were seeded in 24‐well plates at an initial density of 5 × 10^4^ cells/well. They were then cultured in adipogenic medium supplemented with α‐MEM containing 10% FBS, 0.5 mmol/L 3‐isobutyl‐1‐methylxanthine (IBMX; Sigma‐Aldrich, USA), 1 μmol/L hydrocortisone (Sigma‐Aldrich, USA) and 0.1 mmol/L indomethacin (Sigma‐Aldrich) for 14 days with medium changes every 72 hours. After day 14, oil globules were detected by Oil Red O staining (Sigma‐Aldrich).

#### 2.2.3 Chondrogenic differentiation

To induce chondrogenic differentiation, the third passages of GMSCs were seeded in 24‐well plates at an initial density of 1 × 10^5^ cells/well. They were then cultured in chondrogenic medium (STEMPRO Chondrogenesis Differentiation Kit; Gibco) for 14 days with medium changes every 72 hours. After day 14, the presence of cartilage‐specific proteoglycan core protein was detected by Alcian Blue staining (Sigma‐Aldrich).

### 2.3 Flow cytometric analysis

To identify GMSCs, the third passages of GMSCs which were cultured on 60‐mm culture dishes were harvested by .1% trypsin‐EDTA (Sigma‐Aldrich). To quench the enzyme, 1% FBS was added and then the cells were centrifuged at 1000 × *g* for 5 minutes. The cell pellets were resuspended in ice‐cold PBS with 1% FBS. After filtered through a 70‐μm cell strainer (BD Biosciences), the cells were adjusted to a concentration of 1 × 10^7^ cells/mL and separated in Falcon tubes. Then, the cells were incubated in dark at 4°C with specific FITC and PE rat monoclonal antibodies for mouse CD90, CD105, CD45 and CD19 (eBioscience^™^) for 30 minutes. A flow cytometer (FACS Aria II; BD Biosciences) was used to perform flow cytometric analysis.

To compare GMSCs with BMSCs, during osteogenic induction, GMSCs and BMSCs were collected at days 0, 3, 7 and 14. Cells were incubated with anti‐CD90/Thy1 (FITC; eBioscience^™^). A flow cytometer (FACS Aria II; BD Biosciences) was used to perform flow cytometric analysis. Each experiment was performed in three replicates.

### 2.4 Determination of miR‐146a and miR‐155 expression using RT‐qPCR

TaqMan^®^ Pri‐miRNA Assays (miR‐146a assay #468; miR‐155 assay #2571; snoRNA202 assay #1232, Applied Biosystems, Life Technologies) were used to perform reverse transcription of total RNA. After evaluation, cDNA was amplified using the PCR primers and Universal Master Mix II (Applied Biosystems) in a PCR instrument (ABI Prism 7300 Sequence Detection System, Applied Biosystems). According to the manufacturer's instructions, cycling parameters were showed in supporting information Table 1.^17^

The endogenous control (snoRNA202) was used to perform data normalization, and comparisons among samples were made by the comparative CT method.

### 2.5 Cell proliferation

According to the manufacturer's protocol, Cell Counting kit‑8 (CCK‑8; Dojindo Laboratories) was used to detect cell proliferation. GMSCs and BMSCs were seeded into a 96‑well plate at a density of 5 × 10^3^ cells/well, and each well contained 100 μL of the α‐MEM with 10% FBS for culturing cells. At the indicated time‐points (1, 3, 5, 7, 9 days) after seeding, 10 μL CCK‑8 reagent was added to each well and then incubated at the 37℃ for 1 hour. A microplate reader (Wallac 1420 Arvo Sx) was used to measure the absorbance at a wavelength of 450 nm. To eliminate the background, cell medium without cells which was treated with the same assay was used as control. Each experiment was performed in three replicates.

### 2.6 ALP‐positive cell staining

After osteogenic induction, GMSCs and BMSCs were detected by ALP‐positive cell staining solution (Sigma‐Aldrich). The cells were washed twice with PBS and then fixed in 3.7% formalin for 10 minutes. The fixed cells were washed twice with PBS again and then incubated in 1 mL staining solution for 20 minutes at 37°C to identify blue ALP‐positive cells. At last, to stop the staining reaction, the cells were then washed with PBS. A microscope (Biozero BZ‐8000; Keyence) was used to capture digital images.^18^

### 2.7 ALP activity assay

After osteogenic induction, GMSCs and BMSCs were detected for the ALP activity by using a colorimetric p‐nitrophenyl phosphate (pNPP) assay with the ALP detection kit (Wako Chemical). ALP activity was then normalized by the DNA content. Following standard protocols, DNA was quantified using the Quant‐iT PicoGreen Kit (Invitrogen). Each experiment was performed in three replicates, and data were expressed as means ± SD of three replicates.

### 2.8 Alizarin red staining

After osteogenic induction 14 days, GMSCs and BMSCs were detected for mineralized nodules by using Alizarin red staining solution. The staining solution was prepared by dissolving alizarin red S (1%) in 1:100 aluminium hydroxide in water, followed by filtration. The cells were washed twice with PBS and then fixed in methanol for 10 minutes. The cells were then washed with water and incubated with 500 μL of alizarin red S solution per well for 2 minutes. After the mineralized nodules were stained red, the reactions were then stopped by washing with water. A microscope (Biozero BZ‐8000; Keyence) was used to capture digital images.^18^

### 2.9 RT‐qPCR for the expression of osteogenic genes

After osteogenic induction, GMSCs and BMSCs were detected for the expression of four genes related to osteogenesis (ALP, runt‑related transcription factor 2 (RUNX2), osterix and osteocalcin) by RT‐qPCR using primer pairs designed using Primer 3 software showed in supporting infomation Table 2. The cells were pooled and homogenized in TRIzol reagent at days 3, 7 and 14 to extract total RNA. The SuperScript First‐Strand Synthesis System for RT‐PCR (Invitrogen) was used to synthesize cDNA. After evaluation, cDNA was amplified by SYBR Green‐based RT‐qPCR in a PCR instrument (ABI Prism 7300 Sequence Detection System, Applied Biosystems). The endogenous control (GAPDH) was used to perform data normalization, and comparisons among samples were made by the comparative CT method.

### 2.10 Statistical analysis

We performed statistical analyses by using SPSS, version 14.0, for Windows (SPSS Inc.) and analysed the differences between the means by Student's *t*‐test. When a *P* value was ≤ .05, data were considered statistically significant.

## 3 RESULTS

### 3.1 Cell expansion and characterization of gingiva‐derived mesenchymal stem cells

**Figure 1:**
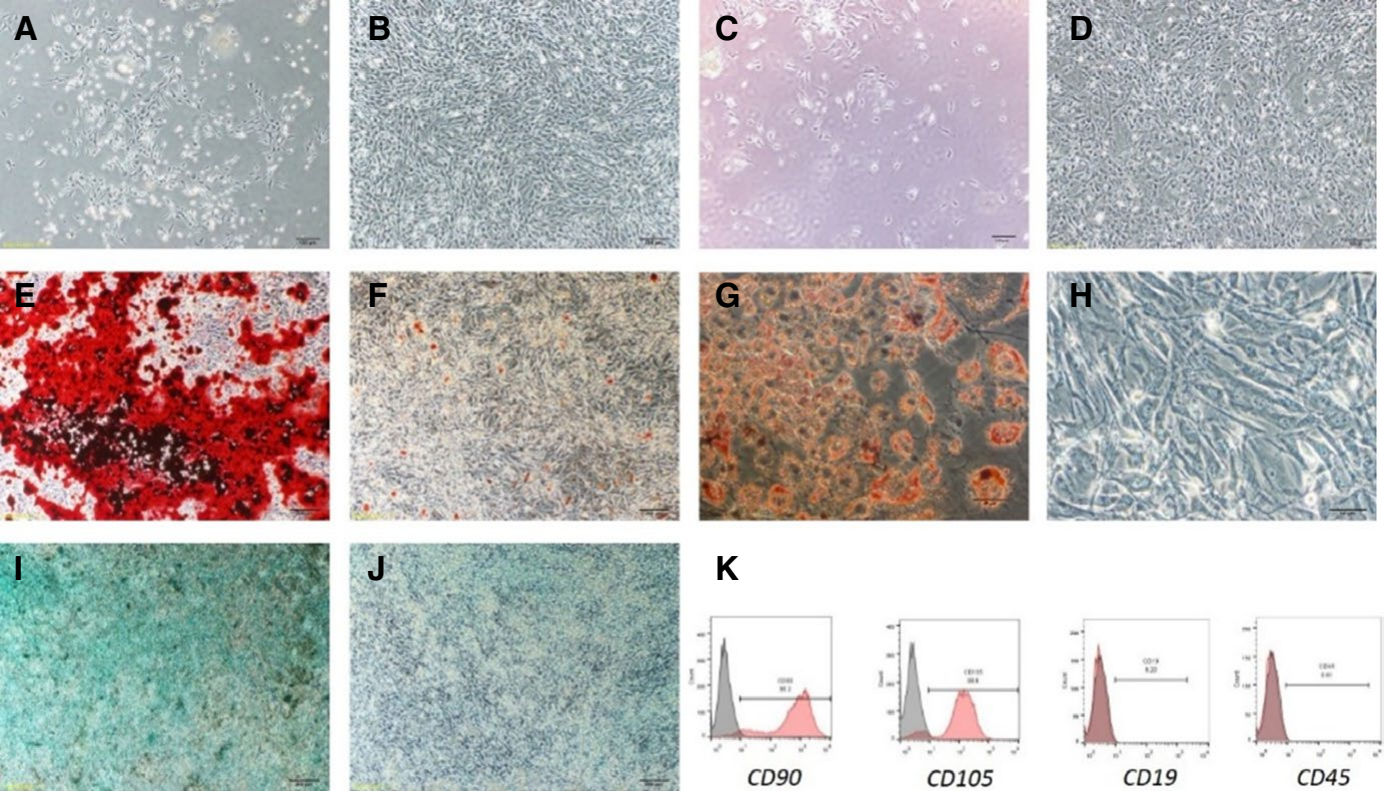
Gingiva‐derived mesenchymal stem cells (GMSCs) characterization. A, The first adherent gingival cells appeared at day 1. The cells showed spindle‐shaped, fibroblast‐like morphology under a light microscope. B, The GMSCs showed high proliferation and formation of colony at day 5. C, The first adherent bone marrow cells appeared at day 5. D, The BMSCs showed high proliferation at day 10. E‐J, showed multi‐lineage differentiation of gingiva‐derived mesenchymal stem cells. E, Osteogenic differentiation at day 21. F, Control at day 21 after Alizarin Red S staining. G, Adipogenic differentiation at day 14. Many oil droplets were observed at day 14. H, Control at day 14 after Oil Red O staining. Oil droplets were not shown at day 14. I, Chondrogenic differentiation at day 14. Chondrogenic differentiation observed after Alcian Blue staining. J, Control at day 14 after Alcian blue. Cell pellet was not formed from the pellet culture. K, Expression of stem cell immunophenotype observed with flow cytometry. The gingiva‐derived mesenchymal stem cells expressed CD90 and CD105 surface antigens, but did not express CD19 and CD45

Once they had been extracted from gingiva, the first adherent cells were observed 1‐2 days after the primary culture. In general, at days 4‐5, the primary cells reached more than 90% confluence. Under microscopy, the gingiva‐derived cells showed spindle‐shaped, fibroblast‐like morphology (Figure 1A,B). While BMSCs grew slowly, the first adherent cells were observed 1‐2 days after the primary culture. In general, at days 8‐10, the primary cells reached more than 90% confluence (Figure 1C,D). The gingiva‐derived cells are able to differentiate into multi‐lineages. After 3 weeks of osteogenic induction, mineralized nodules were stained red by Alizarin Red staining (Figure 1E,F). After 2 weeks of adipogenic induction, many Oil Red O‐positive oil droplets were evident in the cytoplasm of differentiated cells (Figure 1G,H). And after 2 weeks of chondrogenic induction, cartilage‐specific proteoglycan core protein was stained blue by Alcian Blue staining (Figure 1I,J). Moreover, the gingiva‐derived cells were positive for CD90 and CD105 surface antigens, but lacked in the expression of CD19 and CD45 (Figure 1K).

### 3.2 Detection of miR‐146a and miR‐155 by RT‐qPCR

**Figure 2:**
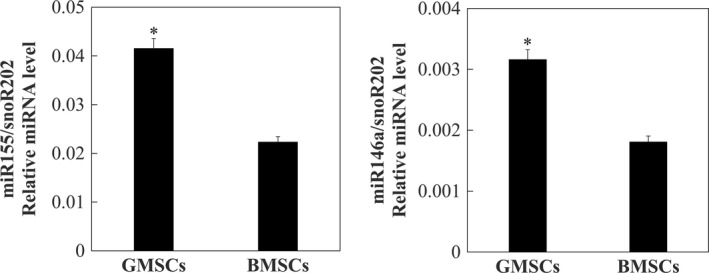
Expression of miR‐146a and miR‐155 in GMSCs and BMSCs. Cells from the third passage were used in this experiment. Data are presented as means and standard errors (n = 3). Detection and quantitation of miR‐155 (left panel) and miR‐146a (right panel) were performed by means of RT‐qPCR using the snoR202 gene as a reference for data normalization. *Significant differences between GMSCs and BMSCs, *P* < .05

Figure 2 showed the result of RT‐qPCR which was detected for the expression of miR‐146a and miR‐155 in GMSCs and BMSCs. This result demonstrated that the expression of miR‐146a and miR‐155 in GMSCs was significantly higher in comparison with BMSCs (*P* < .05).

### 3.3 Cell proliferation

**Figure 3:**
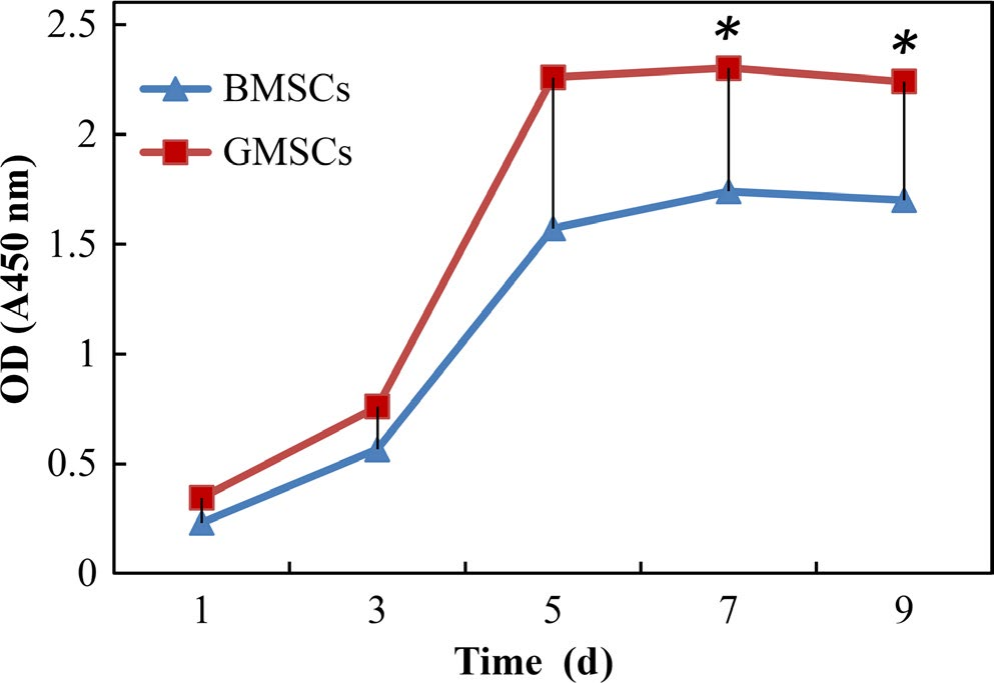
Proliferation rate in the GMSCs and BMSCs. CCK‑8 assay revealed that the GMSCs had a significantly higher cell proliferation rate than BMSCs. *P < .05

The CCK‑8 proliferation assay revealed that the highest significant proliferation was observed at 5 days culture in GMSCs whereas at 7 days culture in BMSCs. At 7 and 9 days culture, there was a significant difference between two groups as the *P*‐value was < .05 (Figure 3). Thus, GMSCs showed higher proliferative capacity than BMSCs.

### 3.4 ALP‐Positive cells and ALP activity

**Figure 4:**
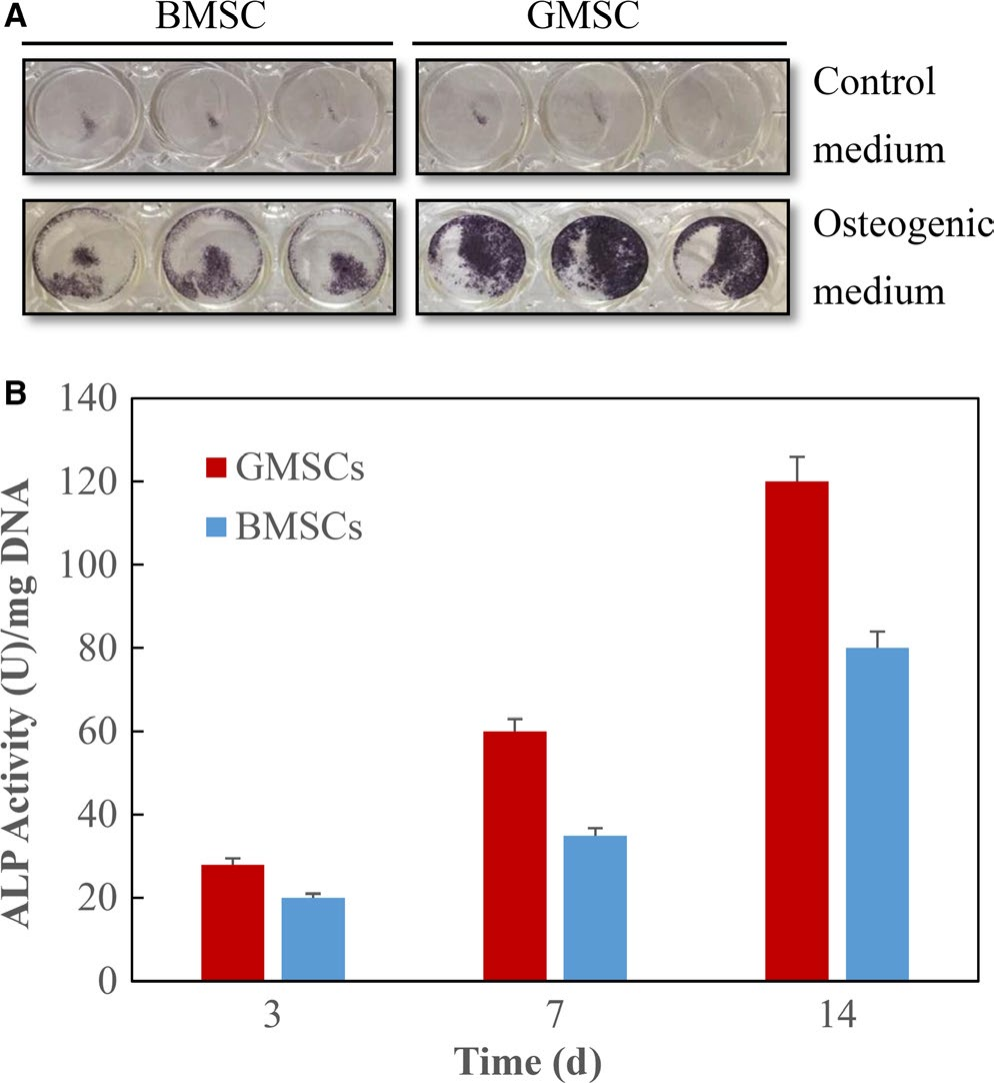
ALP‐positive cells and ALP activity. A, ALP‐positive staining (blue) appeared at day 7 after osteogenic induction. ALP‐positive cells were more prominent in GMSCs compared with BMSCs. B, ALP activity measured by the colorimetric pNPP assay. Each value is mean ± SD; n = 3. The ALP activity was increased significantly over the course of the experiment. At both 7 and 14 d, GMSCs had higher ALP activity than BMSCs (*P* < .05)

GMSCs and BMSCs were cultured in osteogenic medium. After 7 days, ALP‐positive cells were stained by ALP‐positive cell staining solution, and results showed ALP‐positive cells were more prominent in GMSCs compared with BMSCs (Figure 4A). Quantitative ALP activity was measured by normalizing ALP amount to DNA content. The ALP activity of both GMSCs and BMSCs was very low at day 3, increased at day 7 and further increased at day 14. Besides, the ALP activity of GMSCs was significantly higher than that of BMSCs (*P* < .05; Figure 4B).

### 3.5 Mineralized nodule formation

**Figure 5:**
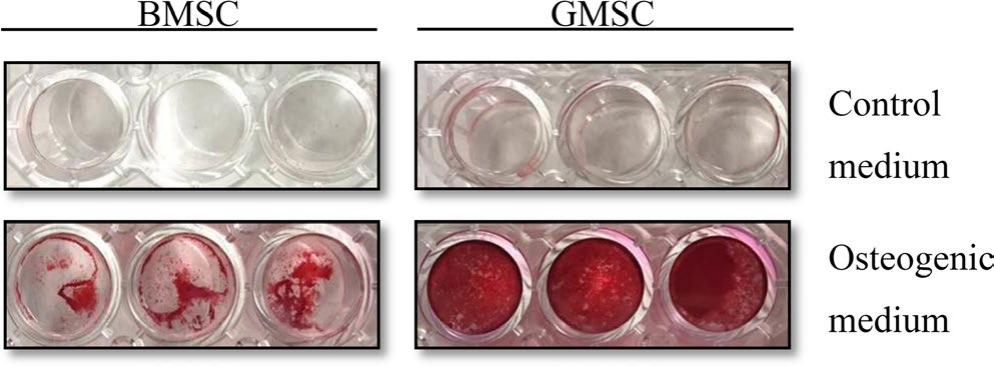
Mineralized nodule formation. Mineralized nodules (red) were observed using Alizarin Red S staining at day 14. GMSCs showed stronger Alizarin Red S staining than BMSCs

As shown in Figure 5, both of GMSCs and BMSCs formed mineralized nodules as stained with Alizarin Red staining. However, GMSCs showed stronger Alizarin Red S staining than BMSCs. This result suggested that osteoblasts generated more calcium deposition in GMSCs.

### 3.6 The expression of osteogenic gene measured by RT‐qPCR

**Figure 6:**
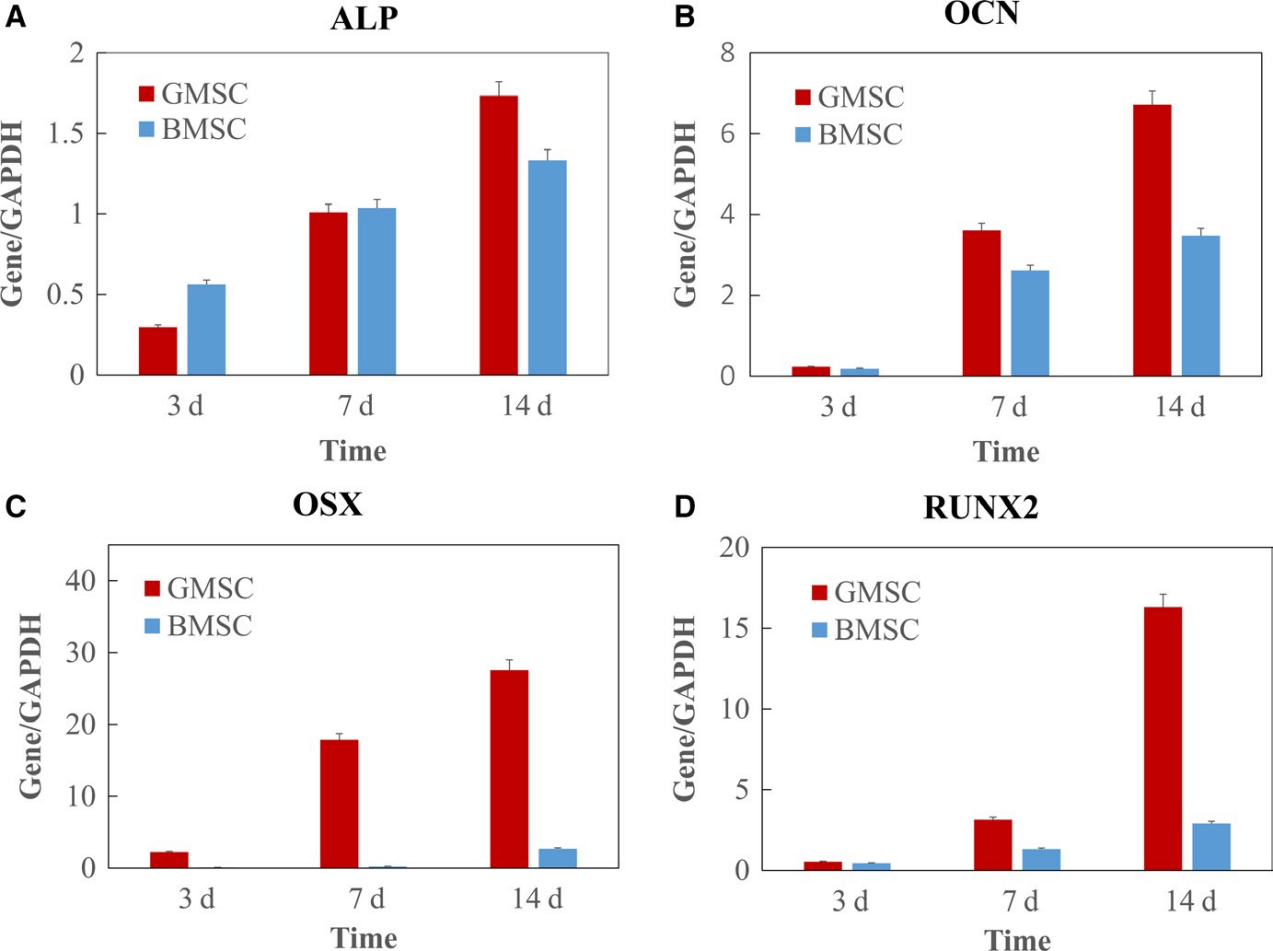
Osteogenic gene expressions measured by RT‐qPCR. The mRNA levels of (A) ALP, (B) OCN, (C) OSX and (D) RUNX2 were measured from total RNA extracted from the GMSCs and BMSCs at days 3, 7 and 14 after osteogenic induction. Data are expressed as mean ± SD; n = 3. GAPDH gene was used as a reference for data normalization. *P* < .01

Osteogenic gene expression was examined. ALP gene expression for both GMSCs and BMSCs was low at day 3, increased at day 7 and greatly increased at day 14. At day 14, GMSCs had significantly higher ALP than BMSCs (*P* < .01; Figure 6A). Osteocalcin, a noncollagenous protein found in bone and dentin, was used as a marker of calcium apposition. OCN gene expression showed a similar trend (Figure 6B). Gene expression of OSX and RUNX2, markers of osteoblast differentiation, was significantly increased in GMSCs compared with BMSCs (Figure 6C,D).

### 3.7 Population of CD90‐positive cells during osteogenic induction in GMSCs and BMSCs

**Figure 7:**
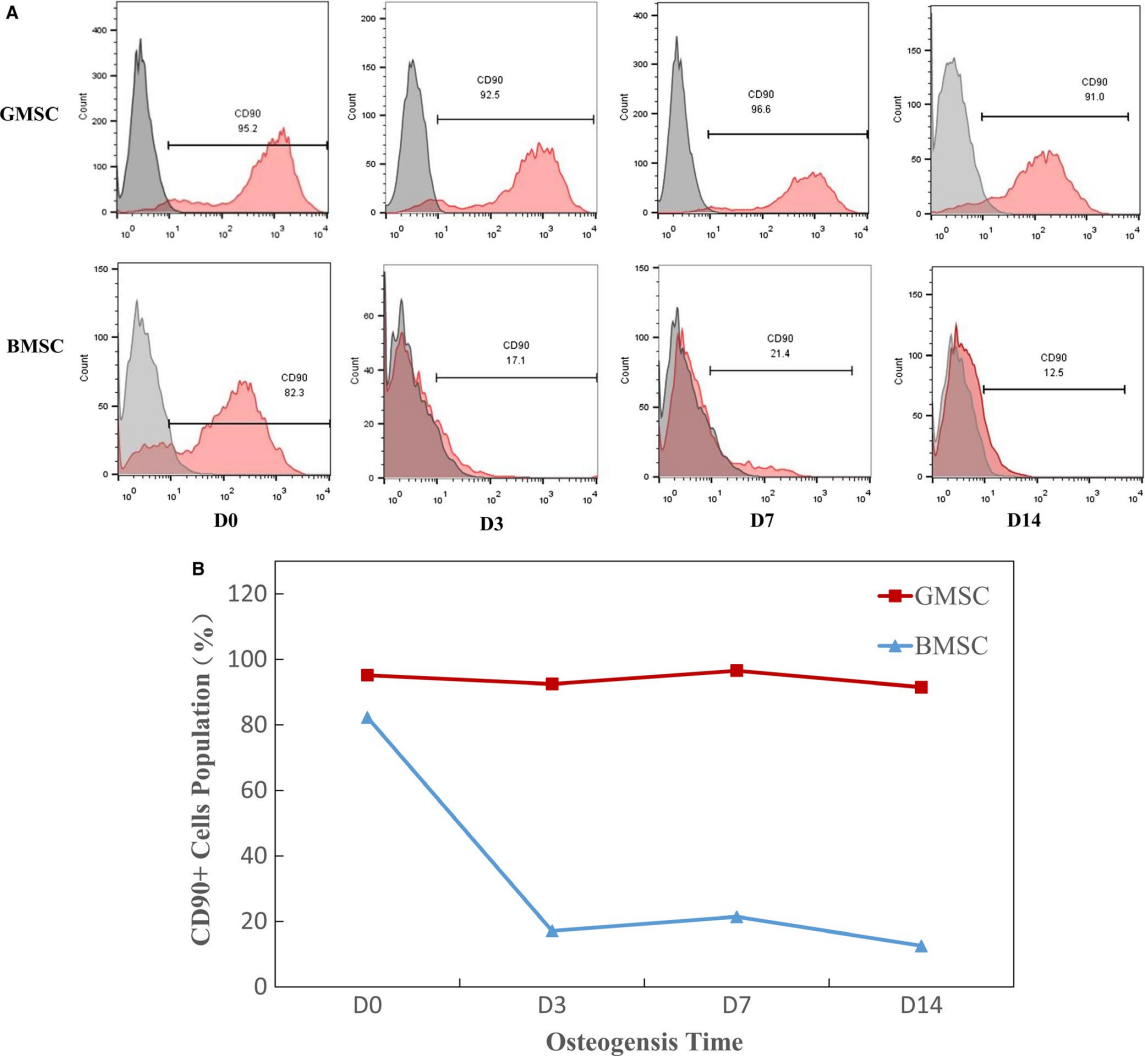
Population of CD90‐positive cells during osteogenic induction in GMSCs and BMSCs. A, Flow cytometric analysis data showed the population of CD90‐positive cells in GMSCs and BMSCs during osteogenic induction. B, number of CD90‐positive cells in GMSCs was significantly higher than that in BMSCs after osteogenic induction

During osteogenic induction, population of CD90‐positive cells at days 0, 3, 7 and 14 in GMSCs and BMSCs was checked by flow cytometric analysis. As shown in Figure 7, the number of CD90‐positive cells stayed very high in GMSCs (around 90%), but in BMSCs the number of CD90‐positive cells decreased drastically during osteogenic induction.

## 4 DISCUSSION

MSCs are present in various organs and have unique properties such as extensive expansion and multi‐lineage differentiation. MSCs’ specific properties make them attractive in tissue engineering.^19^, ^20^, ^21^ In tissue engineering MSCs can be seeded in biocompatible scaffolds and then shaped into anatomical structure. At last to heal the defect the whole structure can be surgically implanted.^22^

Although BM is considered as a main source of MSCs, there are several disadvantages. First of all, extracting BM is a very painful and invasive procedure which can cause donor site morbidity. And only a small percentage of cells in the BM are MSCs. Such low yields require a very large number of bone marrow aspiration.^23^ Even though BMSCs are collected, these cells still need to purify and enrich by several passages in culture because BMSCs are mixed cell populations.^24^ Besides, BMSCs decline in proliferative and differentiation capacity with age and would lose their stem cell characteristics early.^12^

Gingiva is a unique oral reservoir for MSCs which usually contain a large number of stem cells compared to other sources such as bone marrow.^9^ Among oral mucosal tissues, gingiva has specific features. As for tissue biopsy, gingiva is easy to access. Because wound healing is fast and without morbidity. Gingiva surrounds the teeth and is directly attached to the teeth and to the underlying bone. Therefore, compared with the other dental origins, gingiva is the most applicable stem cell source.^25^ Gingival stem cells have similar features of foetal cells with high regeneration potential and also show potent immunoregulatory properties. These features suggest that they may possess specific therapeutic potential. Previous studies showed GMSCs could be used for extraoral tissues wound repair, periodontal regeneration and also peri‐implantitis. Because wound healing in gingiva is fast and without morbidity, nowadays GMSCs have become one of the most exciting alternative for wound repair in extraoral tissues, for example skin wound repair.^26^, ^27^ In a recent study, GMSCs have been used for wound repair by systemic infusion in a mouse model. Their results have proved the treatment of wounds with GMSCs.^13^ One of the mechanisms that GMSCs could improve wound repair is GMSCs are abundant at wound site with multipotent and self‐renewal capabilities. Besides, GMSCs could modulate the local inflammatory response is another mechanism. GMSCs are presumed to promote polarization of macrophages which could increase the level of anti‐inflammatory (IL‐10) and decrease the expression of M1‐cytokines (TNF‐α and IL‐6). Thus, GMSCs could recede the local inflammation, promote angiogenesis and enhance wound repair.^13^ For periodontal tissue regeneration, GMSCs are considered to be a promising cell source. GMSCs could be used in periodontal ligament and alveolar bone regeneration and also the reestablishment of tooth cementum. An earlier in vivo study showed that porcine gingival‐derived stem cells delivered on collagen or inorganic bovine bone matrix with anti‐STRO‐1 antibodies could enhance periodontal regeneration significantly.^28^ This result evidently showed that gingival connective tissue contains multipotent stem cells with a significant periodontal regenerative potential. In a recent in vivo study, GMSCs have been used in a porcine experimental periodontitis model. The result showed a remarkable periodontal regeneration, consisting of newly formed bone, periodontal ligament and cementum.^14^ Peri‐implantitis is the bacterial destructive inflammatory process affecting the soft and hard tissues surrounding and supporting dental implants. Peri‐implantitis is considered to be one of the most serious complications following dental implants.^29^ Recently, one study has tried to apply GMSCs in a peri‐implantitis model in vitro. GMSCs encapsulated in silver lactate‐containing‐loaded alginate hydrogel microspheres showed antimicrobial properties against Aggregatibacter actinomycetemcomitans bacteria in vitro without losing stem cell viability, proliferation and osteogenic differentiation capacity.^30^ This result together with the anti‐inflammatory potential of GMSCs could make GMSCs to be a promising alternative in peri‐implantitis treatment. But further in vivo studies are still needed to confirm this therapeutic potential. Future researches should be focused on systematically comparing gingiva‐derived stem cells to stem cells from other tissues and sources. And the distinct potential of GMSCs should be further confirmed in vitro and in vivo.

To find a better source of MSCs for future therapies of bone defects, we isolated and cultured cells from mouse gingiva and bone marrow, and then compared their proliferation rate and osteogenic differentiation potential.

In our study, we collected gingiva from mouse maxilla following previous research.^31^ According to the previous position paper, defining MSCs needed to meet the following three minimal criteria^32^:

1. Under the standard culture conditions, MSCs should be adherent to plastic.
2. Surface markers should have specific expression: CD105, CD73, CD90 ≥ 90% and CD45, CD34, CD14 or CD11b, CD79α or CD19 and HLA‐DR ≤ 2%.
3. MSCs should have potential of differentiating into osteoblasts adipocytes and chondroblasts.

In our study, the gingiva‐derived cells satisfied minimal criteria. First of all, the gingiva‐derived cells were adherent to plastic. And the gingiva‐derived cells expressed specific surface markers such as CD90 and CD105, but lacked expression of CD19 and CD45. Besides, the gingiva‐derived cells also showed osteogenic, adipogenic and chondrogenic differentiation potential. To further confirm the source of mouse GMSCs are from gingiva, we tried to find proper makers of gingiva. MicroRNAs (miRNAs) are small noncoding RNA molecules which show a tissue‐specific expression pattern. miRNAs play important roles in suppressing protein synthesis and posttranscriptional gene regulation. In a recent study, their results suggested that the expression and regulation of miR‐146a and miR‐155 are more significant in gingiva than in other tissues.^17^ According to this study, we checked the expression of two gingiva‐dependent miRNAs: miR‐146a and miR‐155. And the results showed that the expression of miR‐146a and miR‐155 in GMSCs was significantly higher in comparison with BMSCs. This result proved that our GMSCs were exactly from mouse gingiva.

Compared with BMSCs, GMSCs grew rapidly in vitro expansion. It has shown that the adherent cells isolated from a small piece of gingiva (1‐2 mm^2^) usually reached confluence (60 mm culture dish) after culture for 4 ~ 5 days. The in vitro CCK‐8 proliferation results also showed that the proliferation rate of GMSCs was significantly higher compared with BMSCs. These results have well agreement with previous studies, which showed that GMSCs proliferated faster than BMSCs.^12^

Moreover, we investigated the osteogenic differentiation ability of GMSCs and BMSCs. Osteogenic differentiation was demonstrated by ALP staining, ALP assay and Alizarin red staining. After osteogenic induction, GMSCs showed more ALP stainings at day 7 and ALP activity was significantly higher in gingival group compared with bone marrow group. GMSCs showed stronger Alizarin Red S stainings than BMSCs. This result suggested that osteoblasts generated more mineralized nodules in GMSCs. Osteogenic differentiation was further demonstrated on gene level through the expression of bone‐specific markers, including ALP, Runx2, Osteocalcin (OCN) and osterix (OSX). The results showed that the expression of these four genes was significantly increased in GMSCs compared with BMSCs. The findings of the present study imply that GMSCs showed stronger osteogenesis capacity than BMSCs in vitro.

To further investigate the mechanism in the phenomenon that GMSCs showed higher osteogenic potential than that of BMSCs, we checked the number of CD90‐positive cells during osteogenic induction in GMSCs and BMSCs. CD90 is a mesenchymal stem cell marker,^33^, ^34^ and it could make cells multipotent and promotes osteogenesis.^35^, ^36^, ^37^ As per our previous experimental results, CD90 is a proper surface marker for osteogenesis. It has reported that CD90‐positive selection of human adipose‐derived stem cells enhances their osteogenic potential.^38^ And periosteal stem cells sorted on CD90 expression showed higher potential of proliferative capacity and osteogenic potential in vitro and in vivo compared with unsorted periosteal stem cells.^39^ Recently, it has reported that CD90 is necessary for osteoblast differentiation in vitro and obese CD90 knockout mice demonstrated decreased volume and connectivity of trabecular bone compared with obese wild‐type mice. CD90 deficiency is associated with impaired osteoblastogenesis.^40^ Taken together, these findings establish CD90 is a positive regulator of osteoblast differentiation and modulates bone homeostasis. Findings from our current study showed the number of CD90‐positive cells stayed very high in GMSCs (around 90%), but in BMSCs the number of CD90‐positive cells decreases drastically during osteogenic induction. We can imply that the number of CD90‐positive cells in GMSCs are much higher than in BMSCs is one of the reasons that GMSCs displayed more osteogenesis results than BMSCs.

In summary, we isolated and characterized stem cells from mouse gingiva. GMSCs showed high proliferation and multi‐lineage differentiation capabilities. GMSCs also showed other characteristics of mesenchymal stem cells. Moreover, GMSCs showed stronger osteogenesis capacity than BMSCs in vitro. GMSCs are promising sources for bone defects regeneration. Further research is needed to evaluate the osteogenic potential of GMSCs in vivo.

In conclusion, gingiva can be introduced as a simply accessible source for extraction of mesenchymal stem cells. GMSCs were easy to isolate and proliferated faster than BMSCs without any growth factor. In addition, GMSCs displayed more osteogenesis results than BMSCs. Considering these features, GMSCs may be an outstanding stem cell source for tissue engineering and it could be recommended that GMSCs can be used as a good choice when bone tissue reconstruction is needed.

## CONFLICT OF INTERESTS

The authors declare no competing interests.

## AUTHORS’ CONTRIBUTIONS

Quan Sun, Hidemi Nakata, Maiko Yamamoto and Shinji Kuroda involved in analysing and interpreting data; Quan Sun, Hidemi Nakata and Shinji Kuroda contributed to the conception and design and involved in drafting the article and critical revision; all authors approved the final version to be published.

## ETHICAL APPROVAL

The Institutional Animal Care and Use Committee of Tokyo Medical and Dental University approved the protocol design and procedures (approval number: A2017‐343A).
