# Supplementary material for: Comparison of gingiva‐derived and bone marrow mesenchymal stem cells for osteogenesis
Source: J Cell Mol Med. 2019 Sep 10;23(11):7592–601. doi: 10.1111/jcmm.14632 (PMC6815943; doi:10.1111/jcmm.14632)
Supplement: Supplementary file 1 [file JCMM-23-7592-s001.pdf]

Supporting Information Tables

Table 1. Plate document/experiment parameters for TaqMan® Pri-miRNA Assays.

System	Run	Reaction plate	Plate document/experiment parameters	Thermal cycling conditions		
				Stage	Temp (℃)	Time (mm:ss)
Applied Biosystems 7300/7500 Real-Time PCR System	Standard	96-well standard	Rxn. Volume: 20 µL<br>Ramp Rate: Standard	Hold	50	2:00
				Hold	95	10:00
				Cycle (40 Cycles)	95	0:15
					60	1:00

Table 2. Primer sequences.

Gene	Forward primer (5'→3')	Reverse primer (5'→3')
ALP	CTTCATAAGCAGGCGGGGG	TGCCGATGGCCAGTACTAAA
OCN	GCAATAAGGTAGTGAACAGACTCC	GTTTGTAGGCGGTCTTCAAGC
OSX	CCCACCTAACAGGAGGATTT	CACTGGAATGGAGTGAAACC
RUNX2	GAGGGGGAAATGCCAAATAA	TGCTATTGCCCAAGATTTGC
GAPDH	CCACCCAGAAGACTGTGGAT	CACATTGGGGGTAGGAACAC

OCN, osteocalcin; OSX, osterix; Runx2, runt‑related transcription factor 2.
